# A Review of HIF-1α-Mediated Integration of Metabolic Reprogramming, Mitochondrial Function, and PI3K/Akt–MAPK–Nrf2–NF-κB Signaling

**DOI:** 10.3390/antiox15030378

**Published:** 2026-03-18

**Authors:** Asha Ashraf, Erica D. Bruce

**Affiliations:** Department of Environmental Science, Baylor University, 101 Bagby Ave., Waco, TX 76706, USA; asha_ashraf1@baylor.edu

**Keywords:** hypoxia, hypoxia-inducible factor-1α (HIF-1α), oxidative stress, mitochondrial metabolism, reactive oxygen species (ROS), PI3K/Akt, MAPK, Nrf2, NF-κB

## Abstract

Hypoxia is a common feature of many physiological and pathological conditions, including inflammation, ischemia, and chronic lung disease, where limited oxygen availability disrupts mitochondrial metabolism and promotes excessive reactive oxygen species (ROS) generation. Hypoxia-inducible factor-1α (HIF-1α) is the central transcriptional regulator that enables cellular adaptation to low-oxygen environments by coordinating metabolic reprogramming, mitochondrial remodeling, and redox control. While HIF-1α is widely recognized for its role in promoting glycolysis, evidence indicates that it also suppresses mitochondrial ROS production through coordinated regulation of mitochondrial metabolism, biogenesis, and quality control. This review examines how HIF-1α integrates these mitochondrial and redox-adaptive mechanisms and highlights its bidirectional interactions with key stress-responsive signaling pathways, including PI3K/Akt, MAPK, Nrf2, and NF-κB, that together shape metabolic adaptation, inflammatory responses, and cell survival under hypoxic stress. By integrating these diverse mechanisms, this review provides a comprehensive understanding of the pathophysiology of hypoxia-associated diseases and underscores the therapeutic potential of targeting HIF-1α-regulated metabolic and inflammatory pathways to mitigate oxidative damage induced by hypoxia and environmental stressors.

## 1. Introduction

Oxygen availability is a fundamental determinant of cellular metabolism, survival, and function. In multicellular organisms, fluctuations in oxygen tension occur during normal physiological processes such as development, wound healing, and immune responses [1,2,3], as well as in pathological states including ischemia, inflammation, cancer, and chronic lung disease [4,5,6]. Hypoxia creates a major metabolic and redox challenge by limiting mitochondrial oxidative phosphorylation and increasing ROS production due to inefficient electron transport [6,7]. To survive and adapt under these conditions, cells engage highly coordinated transcriptional and metabolic programs that rewire energy production, redox balance, and stress signaling pathways.

At the center of this adaptive response is hypoxia-inducible factor-1α (HIF-1α), a transcription factor that controls how cells respond to changes in oxygen levels [8]. Under normal oxygen conditions, HIF-1α is quickly broken down, whereas in hypoxia, HIF-1α becomes stable and pairs with HIF-1β, and activates genes that help cells respond to hypoxia [9]. Although HIF-1α is best known for promoting glycolysis and angiogenesis [10], it plays a much broader role. By coordinating changes in metabolism, mitochondrial activity, and redox signaling, HIF-1α helps cells adjust and survive in low-oxygen environments [11,12].

While both HIF-1α and HIF-2α respond to hypoxia, they regulate overlapping yet distinct metabolic programs. HIF-1α primarily drives glycolytic reprogramming, suppression of mitochondrial oxidative metabolism, and induction of antioxidant pathways, thereby coordinating rapid adaptation to oxygen deprivation [13,14]. In contrast, HIF-2α preferentially regulates genes involved in lipid metabolism and erythropoiesis and has been implicated in pathways associated with stemness [15,16,17]. HIF-2α also influences mitochondrial oxidative metabolism in certain contexts through regulation of PPAR-α/PGC-1α–related β-oxidation pathways [18]. Thus, HIF-1α is the principal mediator of hypoxia-induced glycolysis and redox homeostasis, whereas HIF-2α contributes to longer-term metabolic remodeling and tissue-specific responses. Accordingly, the mechanisms discussed in this review largely reflect HIF-1α–dependent regulation.

Mitochondria are both essential energy-producing organelles and major sources of intracellular ROS, particularly under hypoxic conditions where electron transport becomes inefficient [7,19]. Excessive mitochondrial ROS can damage DNA, proteins, and lipids, trigger inflammatory signaling, and activate apoptotic pathways [20,21]. Therefore, effective hypoxic adaptation requires not only maintenance of ATP levels but also strict control of mitochondrial ROS generation. In addition to directly regulating metabolic enzymes, HIF-1α integrates hypoxic signaling with major stress-responsive pathways such as PI3K/Akt, MAPK, Nrf2, and NF-κB. These signaling networks collectively influence cell survival, inflammation, proliferation, and redox homeostasis, and their interaction with HIF-1α enables cells to dynamically adjust to fluctuating oxygen and oxidative stress levels [6,22]. Importantly, understanding this signaling crosstalk provides critical insight into how cells coordinate metabolic adaptation with redox and inflammatory control during hypoxia, and why HIF-1α can exert both protective and regulatory functions across different physiological and pathological contexts.

This review summarizes the current state of knowledge of how HIF-1α suppresses mitochondrial ROS production through multiple regulatory mechanisms, including metabolic reprogramming, inhibition of mitochondrial oxidative metabolism, selective mitophagy, modulation of electron transport chain activity, and reinforcement of antioxidant and NADPH-generating pathways. We also examine the bidirectional interactions between HIF-1α and key stress-responsive signaling pathways—PI3K/Akt, MAPK, Nrf2, and NF-κB—that collectively influence cellular adaptation to hypoxic and oxidative stress. Overall, this review aims to clarify how HIF-1α-mediated metabolic and redox adaptations support cell survival during hypoxia and inflammation thus contributing to the pathophysiology of hypoxia-associated diseases.

## 2. HIF-1α Regulation of Glycolytic Metabolism Under Hypoxia

**Figure 1:**
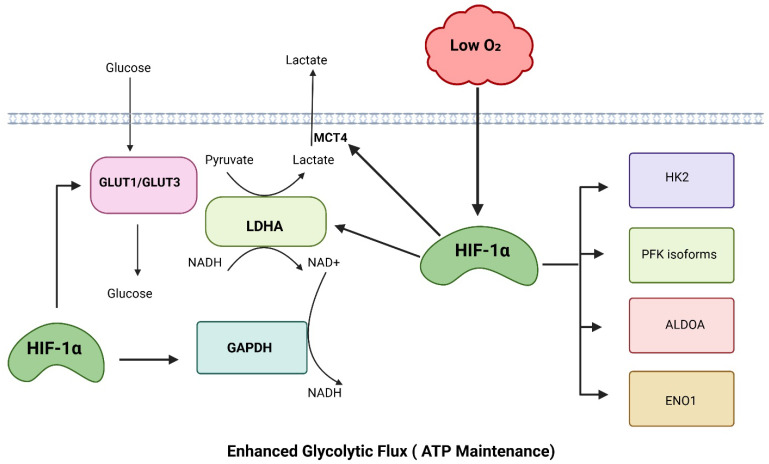
HIF-1α–mediated enhancement of glycolytic flux. Hypoxia stabilizes HIF-1α, promoting glucose uptake and the expression of key glycolytic enzymes, thereby increasing glycolytic flux, lactate production, and ATP maintenance. Created in BioRender. Ashraf, A. (2026) https://BioRender.com/8xmu446 (accessed on 2 February 2026).

Hypoxia-inducible factor-1α (HIF-1α) is a master regulator of cellular metabolic adaptation to low-oxygen environments, primarily by reprogramming glucose metabolism toward glycolysis (Figure 1). This metabolic shift supports ATP production when oxidative phosphorylation becomes limited. HIF-1α binds hypoxia response elements (HREs) and transcriptionally induces glucose transporters (e.g., GLUT1/GLUT3), so the cell imports more glucose per unit time [23]. HIF-1α also transcriptionally induces multiple glycolytic enzymes, including HK2 [24], PFK isoforms [25,26], ALDOA [27], GAPDH [28], ENO1 [29], and LDHA [30], thereby increasing the maximal capacity (Vmax) of the glycolytic pathway and enabling a sufficiently high glycolytic flux to stabilize cellular ATP levels despite the low ATP yield per glucose molecule. In parallel, HIF-1α–mediated induction of LDHA promotes the conversion of pyruvate to lactate, regenerating NAD^+^ from NADH, which is essential to sustain GAPDH activity and prevent glycolytic arrest under hypoxic conditions [31]. The resulting lactate is then exported from the cell by the HIF-inducible transporter monocarboxylate transporter 4 (MCT4) [32], thereby preventing pyruvate buildup. This compensatory mechanism also increases glycolytic flux, maintaining ATP levels even when mitochondrial ATP production falls.

## 3. HIF-1α Regulation of Mitochondrial Function and ROS Production

**Figure 2:**
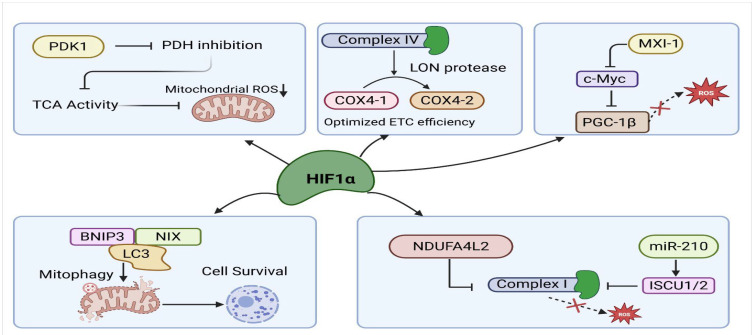
HIF-1α–mediated mechanisms that limit mitochondrial ROS production and promote cell survival under hypoxic stress. HIF-1α reduces mitochondrial ROS production by suppressing TCA cycle activity, optimizing electron transport chain efficiency, promoting mitophagy, and modulating mitochondrial biogenesis and iron–sulfur cluster assembly to support cell survival. Created in BioRender. Ashraf, A. (2026) https://BioRender.com/q1sdtyc (accessed on 2 February 2026).

In addition to promoting glycolytic reprogramming, HIF-1α plays a central role in suppressing mitochondrial oxidative metabolism and reactive oxygen species (ROS) production by coordinating multiple mechanisms of mitochondrial regulation under hypoxic conditions (Figure 2).

### 3.1. HIF-1α Control of TCA Flux and Electron Transport

HIF-1α also actively suppresses mitochondrial TCA cycle activity and oxygen consumption by directly transactivating pyruvate dehydrogenase kinase 1 (PDK1) [13]. PDK1 is a kinase that phosphorylates and inhibits pyruvate dehydrogenase (PDH), preventing pyruvate from entering the mitochondria [33]. Through induction of PDK1, HIF-1α limits carbon flux into the TCA cycle, which is associated with reduced mitochondrial oxygen consumption and is inferred to lower ROS generation under hypoxia. Beyond inducing PDK1, HIF-1α limits mitochondrial ROS production by remodeling of Complex IV, the terminal enzyme of the electron transport chain. Under low oxygen, HIF-1α directly induces transcription of COX4-2. Fukuda et al. showed that HIF-1α induces the hypoxia-adapted COX4-2 subunit and LON protease, leading to replacement of the normoxic COX4-1 subunit in Complex IV [14]. This COX4 isoform switch has been experimentally shown to improve electron transfer efficiency and decrease oxygen consumption, with reduced ROS production inferred from altered electron transport chain activity [14,34]. HIF-1α also reduces mitochondrial ROS production by actively suppressing mitochondrial biogenesis through inhibition of the c-Myc/PGC-1β axis. Under normoxic conditions, c-Myc promotes mitochondrial growth and oxidative metabolism by inducing PGC-1β [35]. PGC-1β is a transcriptional co-activator that drives expression of genes required for mitochondrial biogenesis and electron transport chain assembly. However, during hypoxia, HIF-1α interferes with c-Myc function by promoting MXI-1 expression, a c-Myc antagonist, and by competing with c-Myc for shared coactivators [36,37].

### 3.2. HIF-1α-Mediated Mitophagy

Another major mechanism by which HIF-1α reduces mitochondrial ROS during hypoxia is by activating selective mitophagy through the induction of BNIP3 and NIX (also known as BNIP3L). Supporting this protective role, studies show that HIF-1α–dependent mitophagy limits myocardial infarction in mouse hearts adapted to chronic hypoxia by clearing damaged mitochondria and reducing oxidative injury [38]. These genes contain hypoxia-response elements in their promoters, and HIF-1α strongly upregulates their transcription under oxygen-limited conditions [39]. BNIP3 and NIX are proteins located on the outer mitochondrial membrane that act as receptors for mitophagy. They contain LC3-interacting region (LIR) motifs, which allow them to bind LC3 and recruit autophagosomes [40]. Through this interaction, BNIP3 and NIX help tag damaged or depolarized mitochondria for degradation, ensuring removal of dysfunctional mitochondria during hypoxia [41]. By selectively removing mitochondria that have impaired membrane potential or high electron leak, BNIP3/NIX-mediated mitophagy reduces the burden of ROS-producing organelles and prevents the accumulation of oxidative damage within the cell.

### 3.3. HIF-1α Suppression of Complex I Activity

Under hypoxia, HIF-1α downregulates the expression of several nuclear-encoded subunits of Complex I (NADH dehydrogenase) [42]. Since Complex I is a major site of superoxide generation during impaired electron flow [43], its downregulation significantly lowers ROS output. By simultaneously limiting electron entry at Complex I, HIF-1α shifts mitochondrial respiration into a low-activity state optimized for minimal ROS production. Under hypoxia, HIF-1α induces NDUFA4L2, which inhibits Complex I activity and reduces mitochondrial oxygen consumption, and has been shown to directly decrease mitochondrial ROS levels in cellular models [42]. One study reported that hypoxia enhances cardiac stem cell survival by activating the HIF-1α/NDUFA4L2 pathway, where HIF-1α binds the NDUFA4L2 promoter to reduce apoptosis and decrease caspase-3 activation [44]. Another study showed that hypoxia-induced NDUFA4L2, driven by HIF-1α, promotes osteosarcoma cell survival, migration, invasion, and EMT [45]. In addition to NDUFA4L2, HIF-1α induces miR-210, which targets the iron–sulfur cluster scaffold protein ISCU1/2, thereby impairing Fe–S–dependent Complex I activity and further reducing mitochondrial electron transport and ROS production, as demonstrated by direct measurements of mitochondrial free radical responses under hypoxia [46,47]. Overall, the studies show that HIF-1α suppresses Complex I activity through NDUFA4L2 and miR-210–mediated ISCU repression, thereby reducing mitochondrial ROS and enhancing cell survival under hypoxia.

## 4. HIF-1α and Redox Homeostasis: NADPH and Antioxidant Support

In addition to reshaping glycolysis and suppressing oxidative phosphorylation, HIF-1α promotes a metabolic shift toward reductive glutamine metabolism, a pathway that supports biosynthesis while minimizing mitochondrial ROS production. Under normoxia, glutamine-derived α-ketoglutarate (α-KG) is normally oxidized through the TCA cycle [48], generating NADH [49] that fuel the electron transport chain. However, this oxidative route increases electron flux through Complexes I and III, thereby elevating ROS generation [50]. During hypoxia, HIF-1α induces a reprogramming of glutamine metabolism in which α-KG undergoes reductive carboxylation, mediated primarily by isocitrate dehydrogenase (IDH1/2) [51,52]. This metabolic rerouting reduces the production of mitochondrial NADH, thereby limiting electron input into the ETC and lowering ROS formation [53]. Reductive glutamine metabolism also supports cytosolic NADPH production, further strengthening antioxidant systems.

HIF-1α contributes to redox homeostasis during hypoxia by enhancing pentose phosphate pathway (PPP) activity and maintaining intracellular NADPH levels. HIF-1α increases the expression and activity of key PPP enzymes, including glucose-6-phosphate dehydrogenase (G6PD) and phosphogluconate dehydrogenase (PGD) [54,55], which drive the oxidative branch of the pathway. Studies in rat pheochromocytoma cells and pulmonary artery smooth muscle cells have shown that chronic hypoxia and HIF-1α activation upregulate G6PD expression [56,57]. In a study on gastrointestinal stromal tumors also showed PGD expression markedly upregulated in cells with HIF-1α activation [58]. This shift diverts glucose-6-phosphate away from glycolysis, generating NADPH that fuels major antioxidant systems, which detoxify mitochondrial and cytosolic ROS [59,60]. In addition, HIF-1α promotes the activity of malic enzyme (ME1) and IDH1, two cytosolic NADPH-producing enzymes that further support reductive capacity during oxygen limitation. By maintaining a high NADPH/NADP^+^ ratio, HIF-1α enables cells to regenerate reduced glutathione (GSH), repair oxidized proteins, and prevent oxidative damage [61,62,63]. NADPH serves as the essential electron donor for glutathione reductase and thioredoxin reductase, enabling continuous regeneration of reduced glutathione (GSH) and thioredoxin (Trx) [64,65]. These systems detoxify hydrogen peroxide and lipid peroxides through glutathione peroxidases and peroxiredoxins, thereby preventing accumulation of mitochondrial and cytosolic ROS under hypoxic stress.

These hypoxia-driven metabolic changes are closely coordinated by HIF-1α to maintain redox balance. HIF-1α increases glycolysis while suppressing mitochondrial TCA cycle activity. Through PDK1 induction, pyruvate entry into mitochondria is reduced, and glutamine-dependent reductive carboxylation is promoted [53]. Together, these changes lower mitochondrial NADH production and electron transport activity, which helps reduce ROS generation. At the same time, increased pentose phosphate pathway flux and activation of cytosolic NADPH-producing enzymes support glutathione regeneration and antioxidant defenses [66]. This coordination links glucose and glutamine metabolism to reduced mitochondrial activity in an integrated redox-regulatory network. Supporting these mechanisms, HIF-1α stabilization in a bone regeneration model increased glutaminase-mediated glutathione synthesis and preserved redox homeostasis under metabolic stress [63]. In addition, HIF expression drives glutamine-dependent reductive carboxylation in VHL-deficient tumors in vivo by lowering intracellular citrate levels and promoting α-ketoglutarate reductive flux [67]. Together, these findings show how HIF-1α integrates glycolysis, mitochondrial suppression, and auxiliary pathways to increase NADPH and glutathione production and maintain redox homeostasis during hypoxia.

## 5. HIF-1α–PI3K/Akt Crosstalk

**Figure 3:**
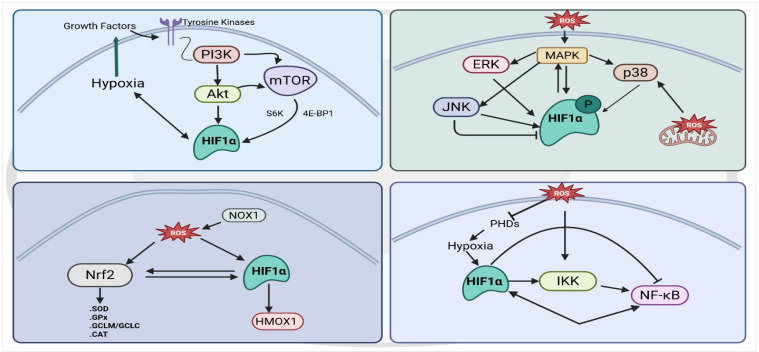
Integrated bidirectional interactions between HIF-1α and major stress-responsive signaling pathways under hypoxic and oxidative stress. HIF-1α interacts with PI3K/Akt–mTOR, MAPK, Nrf2, and NF-κB pathways to integrate hypoxic and redox signals, thereby regulating oxidative stress responses, inflammation, and cell survival. Created in BioRender. Ashraf, A. (2026) https://BioRender.com/y2pskb0 (accessed on 2 February 2026).

HIF-1α and the PI3K/Akt pathway form a tightly interconnected regulatory loop that promotes cell survival and metabolic adaptation under stress [68] (Figure 3). Upstream, PI3K/Akt signaling stabilizes HIF-1α by enhancing mTOR-mediated protein synthesis and by inhibiting prolyl hydroxylases, thereby increasing HIF-1α accumulation even under moderate oxygen conditions [69]. One study shows that mTOR also promotes HIF-1α accumulation by regulating 4E-BP1 and activating S6K, thereby boosting HIF-1α protein levels [70]. Also, several studies have shown that activation of PI3K/Akt reinforces HIF-1α signaling and further increases survival signaling by upregulating genes involved in glucose metabolism [71,72], and also promoting resistance to hypoxia-induced apoptosis [73]. Cheng et al. demonstrated that activation of an Akt–mTOR–HIF-1α axis in β-glucan–trained monocytes drives aerobic glycolysis and enhances cell survival, showing that Akt-dependent induction of HIF-1α–mediated glycolysis strengthens stress-resistant survival programs [74]. HIF-1α also increases expression of growth-promoting targets such as VEGF and IGF-2, which activate receptor tyrosine kinases and further stimulate PI3K/Akt in a feed-forward loop [75,76]. Through this bidirectional crosstalk, HIF-1α and PI3K/Akt cooperatively shift cells toward anabolic metabolism, anti-apoptotic signaling, and mitochondrial protection during hypoxia.

The functional outcome of PI3K/Akt–HIF-1α signaling varies across cell types and disease contexts. In cancer cells, this pathway enhances HIF-1α translation and activity, promoting glycolytic metabolism, VEGF-driven angiogenesis, and tumor growth [77]. PI3K/Akt–HIF-1α signaling regulates glycolytic reprogramming, and disruption of this axis in immune cells reduces LDHA expression and weakens immune function during inflammatory stress [78]. In endothelial cells, PI3K/Akt supports hypoxia-induced HIF-1α and VEGF expression through the VEGFR-2–Akt/eNOS pathway, thereby promoting angiogenic proliferation and vascular remodeling [79]. Similarly, in epithelial cells, PI3K/Akt-mediated HIF-1α stabilization under hypoxic or injury-associated stress drives keratinocyte migration, proliferation, and tissue remodeling through regulation of adhesion molecules such as laminin-332 and growth factor responses, highlighting a key role for this axis in epithelial repair and pathological remodeling [80].

## 6. HIF-1α–MAPK Interdependence in Hypoxic and Stress Responses

HIF-1α is functionally intertwined with MAPK signaling through a bidirectional regulatory relationship, in which MAPK activation modulates HIF-1α stability and transcriptional activity, and HIF-1α, in turn, shapes MAPK-dependent cellular responses under oxidative and hypoxic stress (Figure 3). Mylonis, I. et al. showed that phosphorylation of HIF-1α at Ser641/643 by MAPK promotes its nuclear accumulation and transcriptional activity by preventing CRM1-dependent nuclear export [81]. ERK and p38 MAPK enhance HIF-1α transcriptional activity and stability by phosphorylating HIF-1α or its co-activators [82]. Evidence from endothelial cell studies indicates that hypoxia activates ERK and that ERK1-mediated phosphorylation of HIF-1α is necessary for maximal HIF-1 transcriptional function [83]. Mitochondrial ROS also activate p38 MAPK under hypoxia, and this p38 signaling promotes HIF-1α stabilization and enhances HIF-1–dependent transcription [84,85]. Although JNK activation during hypoxia is context-dependent, one study in neuronal cells showed that JNK inhibition increases HIF-1α and enhances survival [86], whereas another study in cardiomyocytes demonstrated that hypoxia-induced ROS activate JNK to upregulate HIF-1α and drive c-Jun/ATF-2–mediated fibrotic gene expression [87]. Conversely, evidence indicates that HIF-1α can function upstream of MAPK signaling. HIF-1α promotes osteoclast differentiation in RAW264.7 cells through MAPK pathway activation [88], and in hypoxic HSC-T6 cells, increased HIF-1α enhances MAPK-dependent cytoskeletal remodeling and fibrotic gene induction [89]. Together, this bidirectional control ensures that MAPK pathways respond appropriately to hypoxic stress while preventing excessive ROS-driven apoptosis.

## 7. Redox Signaling Links Between HIF-1α and Nrf2

HIF-1α and Nrf2 function as coordinated regulators of cellular redox homeostasis, each capable of enhancing the other’s protective role (Figure 3). Nrf2 is a transcription factor that drives the expression of antioxidant defense genes, enabling cells to manage and neutralize reactive oxygen species. Leacher et al. study showed that NRF2 directly binds and activates an antioxidant-response element upstream of the HIF1A gene, revealing that Nrf2 can transcriptionally upregulate HIF-1α under oxidative stress [90]. In another study, Nrf2 deficiency in kidney tubular epithelial cells reduced HIF-1α activation and without Nrf2, hypoxia-induced HIF-1α protein accumulation, HIF-1α–dependent metabolic gene expression, and downstream targets like HMOX1 were all markedly impaired [91]. Across breast cancer models, NRF2 loss disrupts hypoxia-driven HIF-1α activation and suppresses glycolysis, PPP activity, and autophagy [92], while complementary evidence shows that NRF2 promotes tumor progression by enhancing glycolysis through HIF-1α co-activation and upregulation of key glycolytic enzymes [93]. A separate study shows Arsenic exposure activates Nrf2, which in turn drives HIF-1α activation and cooperatively reprograms cells toward glycolysis and a cancer stem–like phenotype [94].

Although direct transcriptional control of Nrf2 by HIF-1α is not well established, HIF-1α clearly modulates NRF2 activity in a context-dependent manner. In renal tubular cells, HIF-1α activation can either enhance or suppress NRF2 nuclear localization and antioxidant signaling depending on nutrient status [95], while in endothelial cells HIF-1 induction has been shown to downregulate Nrf2 expression and attenuate Nrf2-dependent IL-8 transcription [96]. Under intermittent hypoxia, NOX1-derived ROS co-activate both NRF2 and HIF-1α, with NRF2/Trx1 signaling further augmenting HIF-1α activity [97,98]. This underscores a bidirectional and redox-sensitive crosstalk between these pathways.

The magnitude of reactive oxygen species shapes NRF2 signaling: moderate oxidative stress induces antioxidant genes (HO-1, NQO1), whereas higher ROS shifts responses toward stress regulators such as Klf9 [99]. Acute hypoxia increases NRF2 protein levels, NRF2/ARE binding activity, and antioxidant gene expression, while HIF-1α knockdown reduces NRF2 accumulation and nuclear activity, indicating that HIF-1α supports NRF2 activation under hypoxic stress [100]. In colon cancer, loss of NRF2 prevents hypoxic HIF-1α accumulation, reduces VEGF expression, and limits tumor angiogenesis, highlighting NRF2 as a key regulator of HIF-1α–dependent vascular growth [101]. In glioblastoma, hypoxia-induced NRF2 activation drives NIX-dependent mitophagy, clearing mitochondrial ROS and sustaining HIF/mTOR signaling to support tumor survival in hypoxic niches [102]. However, in certain contexts, their activities are inversely related; for example, fumarate-induced NRF2 activation suppresses HIF-1α, whereas NRF2 depletion further elevates HIF-1α levels [103]. In innate immune cells, loss of BMAL1 reduces NRF2 activity, leading to increased ROS accumulation, enhanced HIF-1α stabilization, and exaggerated inflammatory cytokine production [104]. Likewise, in alcoholic liver injury, NRF2 deficiency enhances HIF-1α activation and steatosis, whereas NRF2 activation suppresses HIF-1α signaling and protects against lipid accumulation [105].

## 8. HIF-1α and NF-κB: A Bidirectional Regulatory Axis

The NF-κB pathway and HIF-1α are tightly interconnected regulators of inflammation and hypoxic stress (Figure 3). Multiple studies demonstrate that NF-κB directly enhances HIF-1α transcription. van Uden et al. [106] showed that NF-κB subunits bind the HIF-1A promoter, and that TNF-α–induced NF-κB activation increases HIF-1α mRNA under normoxia, while NF-κB depletion suppresses basal HIF-1α levels. Consistent with this, Rius et al. [107] demonstrated that IKKβ-dependent NF-κB activation is required for HIF-1α mRNA expression in macrophages, with ChIP assays confirming RelA binding to a functional κB site in the HIF1A promoter. In cancer cells, Yoshida et al. [108] reported that overexpression of NF-κB/p65 increases HIF-1α promoter activity and protein expression. Earlier work by Frede et al. [109] showed that LPS induces NF-κB–dependent upregulation of HIF-1α and HIF-1 target genes in monocytes even under normoxia, demonstrating that inflammatory NF-κB activation can generate a pseudo-hypoxic response. Together, these findings establish NF-κB as a potent transcriptional activator of HIF-1α across immune and cancer cell contexts.

Beyond being transcriptionally regulated by NF-κB, HIF-1α can actively enhance NF-κB signaling. Walmsley et al. [110] identified NF-κB as a hypoxia-regulated, showing that hypoxia-induced neutrophil survival requires HIF-1α–dependent NF-κB activation. Another study in the epidermis by Scortegagna et al. showed that epithelial HIF-1α is required for full NF-κB activation and pro-inflammatory gene expression, indicating that HIF-1α can drive NF-κB–dependent epithelial inflammation [111]. Several studies show that hypoxia itself activates NF-κB: Koong et al. [112] reported that hypoxia induces tyrosine phosphorylation of IκBα to activate NF-κB, while Culver et al. [113] found that hypoxia stimulates IKK through a CaMK2-dependent mechanism to drive NF-κB activation independently of inflammation. Collectively, these findings establish that HIF-1α not only responds to NF-κB but also reinforces NF-κB signaling.

Despite these activating interactions, growing evidence indicates that HIF-1α can function as a negative regulator of NF-κB–driven inflammation. A study by Bandarra et al. [114] demonstrated that loss of HIF-1α enhances NF-κB transcriptional activity and increases expression of NF-κB target genes. Consistent with this anti-inflammatory role, Okada et al. [115] showed that HIF-1α maintains articular cartilage homeostasis by suppressing NF-κB activation and reducing catabolic gene expression in chondrocytes. Additional mechanistic support comes from Scholz et al. [116], who reported that inhibition of HIF-hydroxylases, which stabilizes HIF-1α, suppresses IL-1β-induced NF-κB activity, linking hypoxia–HIF-1α signaling to reduced NF-κB responsiveness during inflammatory stress. A broader analysis from D’Ignazio et al. [117] further highlights that HIF-1α can dampen NF-κB signaling across multiple immune and stromal cell types, acting as a counter-regulatory transcription factor to prevent excessive inflammatory activation. Overall, the evidence reveals a bidirectional and context-dependent relationship in which HIF-1α not only amplifies NF-κB signaling during hypoxic or inflammatory activation but can also restrain NF-κB-driven responses to maintain inflammatory balance.

Studies indicate that reactive oxygen species exert bidirectional effects on NF-κB signaling and moderate ROS promote NF-κB activation and pro-survival signaling, whereas excessive ROS impair NF-κB activity through oxidative stress-mediated inhibition [118,119]. Hypoxia activates NF-κB via oxygen-sensing prolyl hydroxylases, which regulate IKK activity and couple low oxygen tension to NF-κB signaling [120]. In colon cancer models, inhibition of HIF-1α fails to fully suppress angiogenesis due to compensatory NF-κB–dependent induction of IL-8 driven by hypoxia-associated ROS, highlighting cooperative and redundant pro-angiogenic signaling between HIF-1α and NF-κB pathways [121]. In pulmonary artery smooth muscle cells, ROS generated by H_2_O_2_ or NOX4-containing NADPH oxidase activate NF-κB and subsequently induce HIF-1α transcription, demonstrating a direct redox-dependent link between NF-κB and HIF-1α signaling [122]. Another study in hypoxic lung endothelium, erythrocyte-derived H_2_O_2_ acts as a signaling mediator that drives NF-κB (p65) nuclear translocation and HIF-1α stabilization, thereby initiating pro-inflammatory vascular gene expression [123].

## 9. Conclusions

HIF-1α functions as a central integrator of metabolic, mitochondrial, and redox adaptation during hypoxia. Beyond its well-known role in promoting glycolysis, HIF-1α helps limit mitochondrial ROS production by coordinating changes in mitochondrial metabolism and strengthening cellular antioxidant defenses. These mechanisms are further shaped by interactions between HIF-1α and key stress-responsive signaling pathways, including PI3K/Akt, MAPK, Nrf2, and NF-κB, allowing cells to balance energy demands, redox homeostasis, and inflammatory responses under hypoxic stress. Understanding how HIF-1α coordinates these responses helps explain hypoxia-related diseases and identifies metabolic and redox pathways as potential therapeutic targets to reduce oxidative damage and inflammation.

## Data Availability

No new data were created or analyzed in this study. Data sharing is not applicable to this article.
